# Urolithin B, a Gut Microbiota Metabolite, Reduced Susceptibility to Myocardial Arrhythmic Predisposition after Hypoxia

**DOI:** 10.1155/2022/6517266

**Published:** 2022-02-08

**Authors:** Xin Huang, Hong Gao, Xiaojie Jiang, Zeqi Zheng

**Affiliations:** ^1^Department of Cardiology, The First Affiliated Hospital of Nanchang University, Nanchang 330008, China; ^2^Department of Cardiology, The First Hospital of Nanchang, The Third Affiliated Hospital of Nanchang University, Affiliated Hospital of Sun Yat-sen University, Nanchang 330008, China

## Abstract

Cardiomyocyte apoptosis, neural remodeling, and gap junction channel change play critical roles in ventricular arrhythmia (VA) after acute myocardial infarction (AMI). Urolithin B (UB), one of the gut metabolites of ellagitannins, a class of antioxidant polyphenols, has various biological activities, but its direct role in cardiomyocyte apoptosis, neural remodeling, and gap junction channel change after AMI remains elusive. We investigated whether urolithin B reduced susceptibility of myocardial arrhythmic after myocardial infarction (MI). In vitro, the cardiomyocytes were subjected to hypoxia (94% N_2_/5% CO_2_/1% O_2_) for 3 hours. Cardiomyocyte apoptosis was assessed by TUNEL staining and western blotting. Urolithin B was found to decrease the number of apoptotic cells after hypoxia. Moreover, there was a substantial decrease in the expression of neural remodeling markers in the urolithin B treatment group. Urolithin B significantly increased the expression level of gap junction channel protein. Mechanistically, urolithin B inhibited cardiomyocyte apoptosis by activating Akt/the mammalian target of rapamycin (mTOR) pathway, and the protection of urolithin B against cardiomyocyte apoptosis was compromised with Akt gene silencing. Furthermore, urolithin B suppressed nuclear translocation of nuclear factor-kB (NF-*κ*B) to facilitate nerve remodeling. Taken together, our findings suggested that UB reduced the occurrence of myocardial arrhythmias after hypoxia via regulation of the Akt/mTOR pathway and NF-*κ*B nuclear translocation, which highlights the potential of UB as a novel therapy for ischemic heart disease.

## 1. Introduction

Acute myocardial infarction (AMI), the leading cause of death worldwide, imposes immense health and economic burden [1, 2]. Arrhythmia, especially ventricular arrhythmia, is the major cause of sudden death in AMI patients [3]. Sympathetic neural remodeling [4, 5], gap junction remodeling [3], and cardiomyocyte apoptosis [6] contribute to fatal ventricular arrhythmia. In patients with AMI, timely and effective myocardial reperfusion using either thrombolytic therapy or primary percutaneous coronary intervention is one of the main treatment options against ventricular arrhythmia [2]. However, increasing numbers of evidence indicate that pharmacological interventions targeting neural remodeling, gap junction channel remodeling, and cardiomyocyte apoptosis reduce the incidence of arrhythmia, improving long-term prognosis. As a result, the regulation of neural remodeling, gap junction channels, and cardiomyocyte apoptosis via medications may be an essential therapeutic strategy for attenuating MI-induced arrhythmia.

Ellagitannins (ETs) are a class of polyphenols enriched in pomegranates, walnuts, and berries [2]. ETs are hydrolyzed by the gut microbiota to ellagic acid (EA), which is further hydrolyzed into urolithins [7]. Urolithin A (UA) and urolithin B (UB) are the main components of urolithin detected in various tissues [8]. Previous research demonstrated that UB protected the apoptosis [2], inflammation [9], and neural remodeling of myocardial tissues and inhibited gap junction channel expression, which were the key factors that cause arrhythmia [10, 11]. However, the mechanism of urolithin B in reducing the occurrence of arrhythmia through cardiomyocyte apoptosis, neural remodeling, and inhibited gap junction channels is unclear to date.

In this study, we investigated the relationship between the role of urolithin B and cardiomyocyte apoptosis, neural remodeling, and gap junction channels.

## 2. Investigations and Results

### 2.1. Ischemia Stimulates Cell Apoptosis and Alters Gap Junction and Neural Remodeling Levels of Cardiomyocyte In Vivo

**Figure 1:**
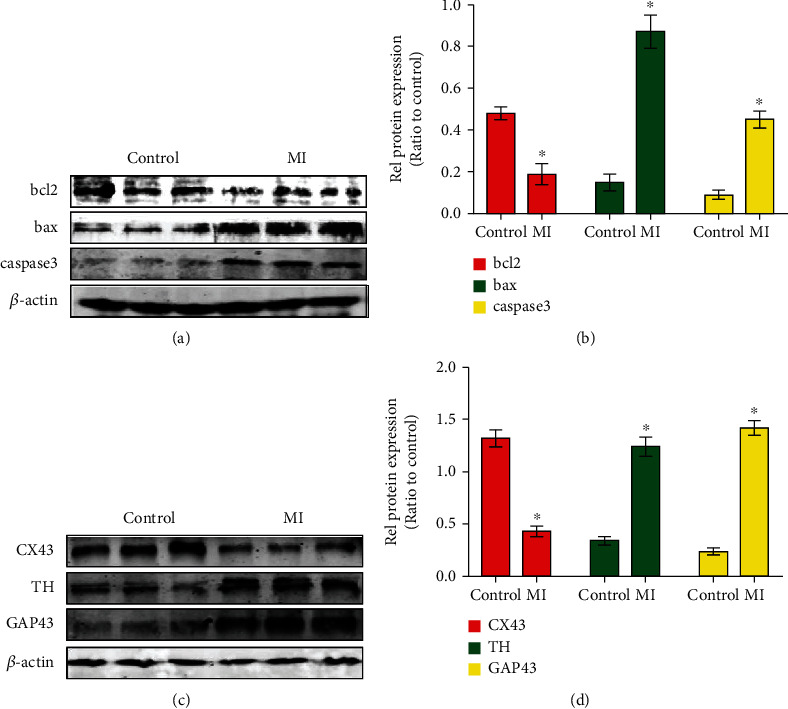
Ischemia stimulates cell apoptosis and alters gap junction and neural remodeling levels in vivo. (a, b) Western blotting and densitometric analysis of the protein levels of bcl2, bax, and caspase-3 in myocardial infarction tissues compared with control tissues. ^∗^*p* < 0.05; *n* = 5 per group (Student's *t*-test). (c, d) Western blotting and densitometric analysis of the protein levels of CX43, TH, and GAP43 in myocardial infarction tissues compared with control tissues. ^∗^*p* < 0.05; *n* = 5 per group (Student's *t*-test).

To investigate the impact of ischemia on apoptosis, neural remodeling, and gap junction change in acute myocardial infarction tissues, we first examined the expression of apoptosis protein, neural remodeling protein, and gap junction protein in mouse myocardial infarction and normal heart samples. Western blotting results revealed that the expression of the apoptosis markers, bax and caspase-3, was substantially increased in myocardial infarction tissues compared with control tissues. By contrast, bcl2, an antiapoptotic marker, was lower than that in control tissues (Figures 1(a) and 1(b)). In addition, the suppressed gap junction protein connexin 43 (Cx43) was found in myocardial infarction tissues. Meanwhile, growth-associated protein-43 (GAP-43), a marker of nerve sprouting, and tyrosine hydroxylase (TH), a marker of the sympathetic nerve, were significantly elevated in myocardial infarction mice (Figures 1(c) and 1(d)). Taken together, these results showed that ischemia increased apoptosis and neural remodeling and downregulated gap junction of the cardiomyocyte in the myocardial infarction mice.

### 2.2. Hypoxia Stimulates Cardiomyocyte Apoptosis and Alters Gap Junction and Neural Remodeling Levels In Vitro

**Figure 2:**
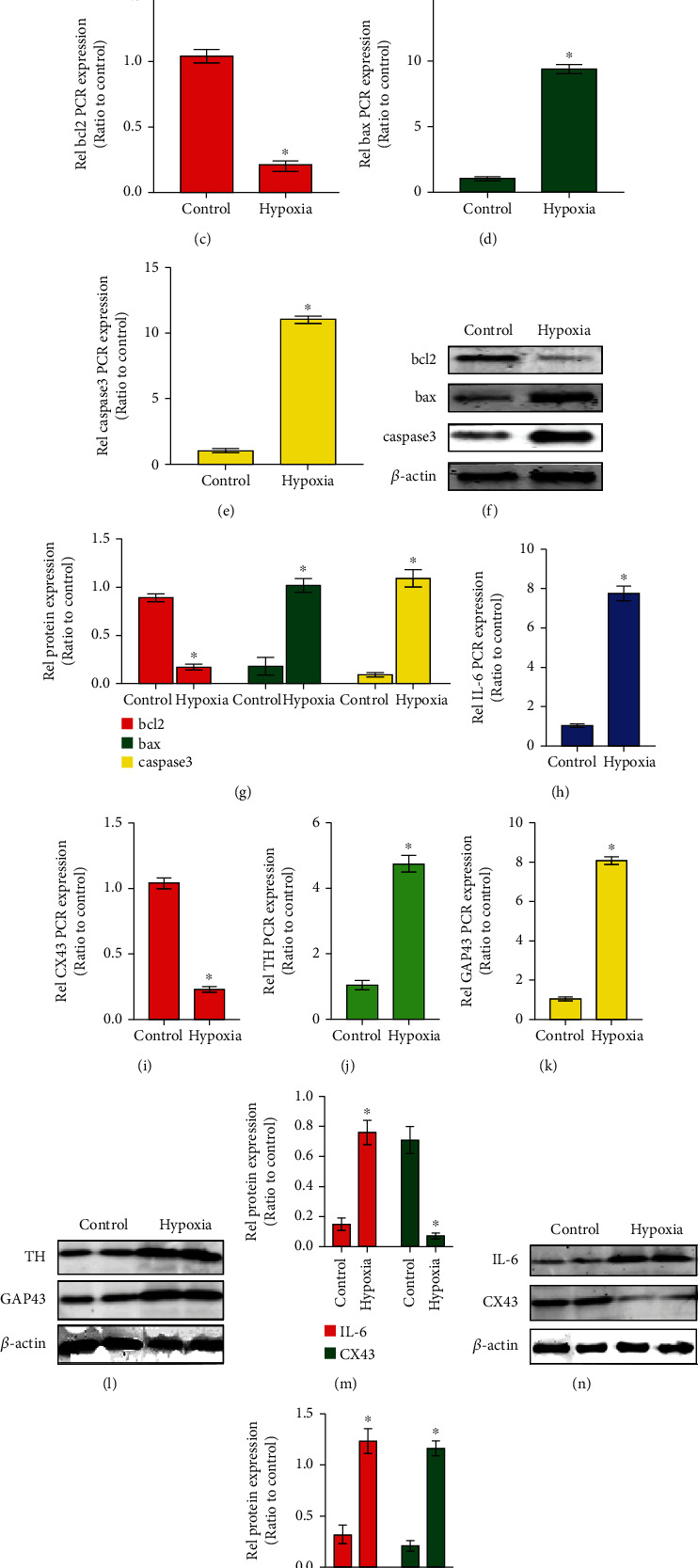
Hypoxia stimulates cardiomyocyte apoptosis and alters gap junction and neural remodeling levels in vitro. (a) TUNEL immunofluorescence staining in cardiomyocytes in the hypoxia group and control group (bar = 50 *μ*m). (b) Quantification of the percentage of TUNEL-positive cardiomyocytes in the hypoxia group and control group. ^∗^*p* < 0.05; *n* = 5 per group (Student's *t*-test). (c–e) Q-PCR assay detecting the expression levels of bcl2, bax, and caspase-3 in the hypoxia group and control group. (f) WB assay detecting the expression levels of bcl2, bax, and caspase-3 in the hypoxia group and control group. (g) Quantification of bcl2, bax, and caspase-3 protein levels. ^∗^*p* < 0.05; *n* = 5 per group (Student's *t*-test). (h–k) Q-PCR assay detecting the expression levels of IL-6, CX43, TH, and GAP43 in the hypoxia group and control group. (l–o) Western blotting and densitometric analysis of the protein levels of IL-6, CX43, TH, and GAP43 in the hypoxia group and control group. ^∗^*p* < 0.05; *n* = 5 per group (Student's *t*-test).

We further confirmed the effect of hypoxia on apoptosis protein, neural remodeling protein, and gap junction protein expression in cardiomyocytes. The TUNEL assay showed that hypoxia upregulated the apoptotic rate of cardiomyocytes compared with the control group (Figures 2(a) and 2(b)). Furthermore, the qRT-PCR and western blotting results suggested that the mRNA and protein expression of Bax, caspase-3, IL-6, TH, and GAP43 was increased in the hypoxia group, while the expression of bcl2 and CX43 was downregulated in the hypoxia group, compared with the control group (Figures 2(c)–2(o)).

### 2.3. Urolithin B Reduced Myocardial Apoptosis and Regulated Neural Remodeling and Gap Junction Channels

**Figure 3:**
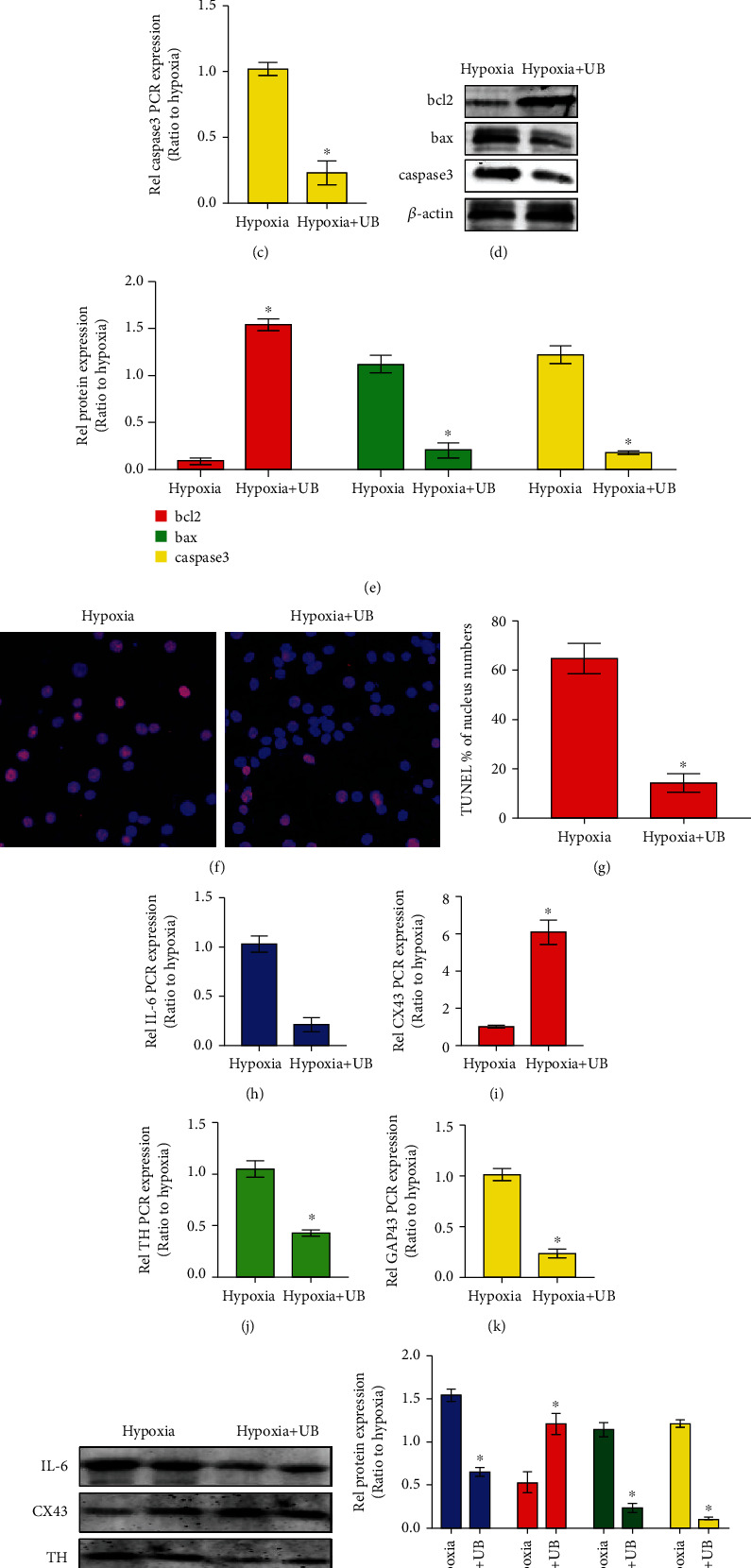
Urolithin B reduced myocardial apoptosis and regulated neural remodeling and gap junction channels. (a–c) Q-PCR assay detecting the expression levels of bcl2, bax, and caspase-3 in the hypoxia group and the hypoxia+urolithin B group. (d, e) WB assay detecting the expression levels of bcl2, bax, and caspase-3 in the hypoxia group and the hypoxia+urolithin B group. (f) TUNEL immunofluorescence staining in cardiomyocytes in the hypoxia group and the hypoxia+urolithin B group (bar = 50 *μ*m). (g) Quantification of the percentage of TUNEL-positive cardiomyocytes in the hypoxia group and the hypoxia+urolithin B group. ^∗^*p* < 0.05; *n* = 5 per group (Student's *t*-test). (h–k) Q-PCR assay detecting the expression levels of IL-6, CX43, TH, and GAP43 in the hypoxia group and the hypoxia+urolithin B group. (l, m) Western blotting and densitometric analysis of the protein levels of IL-6, CX43, TH, and GAP43 in the hypoxia group and control group. ^∗^*p* < 0.05; *n* = 5 per group (Student's *t*-test).

Previous work has demonstrated that urolithin B prevented cardiomyocytes from apoptosis caused by hypoxia [2]. We detected alterations in bcl2, bax, and caspase-3 and apoptotic rate of cardiomyocytes between the hypoxia group and the hypoxia+urolithin B group. The mRNA expression of bcl2 was markedly increased in the cardiomyocyte in the hypoxia+urolithin B group, while the expression of bax and caspase-3 was decreased in the hypoxia+urolithin B group (Figures 3(a)–3(c)). The protein expression patterns of bcl2, bax, and caspase-3 identified by western blotting were concordant with those of the qRT-PCR assay (Figures 3(d) and 3(e)). TUNEL staining suggested that the apoptotic rate of cardiomyocytes was dramatically downregulated in the urolithin B treatment group (Figures 3(f) and 3(g)). Previous research indicated that urolithin B retards the expression of the inflammatory cytokine IL-6 [9], which influences the expression of the gap junction protein Cx43 and neural remodeling proteins GAP-43 and TH [10, 11]. Therefore, we explored whether urolithin B regulates cardiomyocyte Cx43, GAP-43, and TH via IL-6. We determined the expression level of IL-6, Cx43, GAP-43, and TH by qRT-PCR. As expected, there was a substantial decrease in the mRNA expression of IL-6, GAP-43, and TH in the hypoxia+urolithin B group compared with the hypoxia group, while urolithin B significantly increased the level of Cx43 (Figures 3(h)–3(k)). Western blotting revealed that the expression trend of bcl2, bax, and caspase-3 proteins was consistent with qRT-PCR results. Based on these results, urolithin B reduced susceptibility of myocardial arrhythmia after hypoxia via suppressing cardiomyocyte apoptosis, inhibiting neural remodeling, and promoting gap junction channel expression.

### 2.4. Urolithin B Activates the Akt/mTOR Pathway to Inhibit Hypoxia-Induced Cardiomyocyte Apoptosis and Suppresses Nuclear Translocation of NF-*κ*B to Facilitate Nerve Remodeling

**Figure 4:**
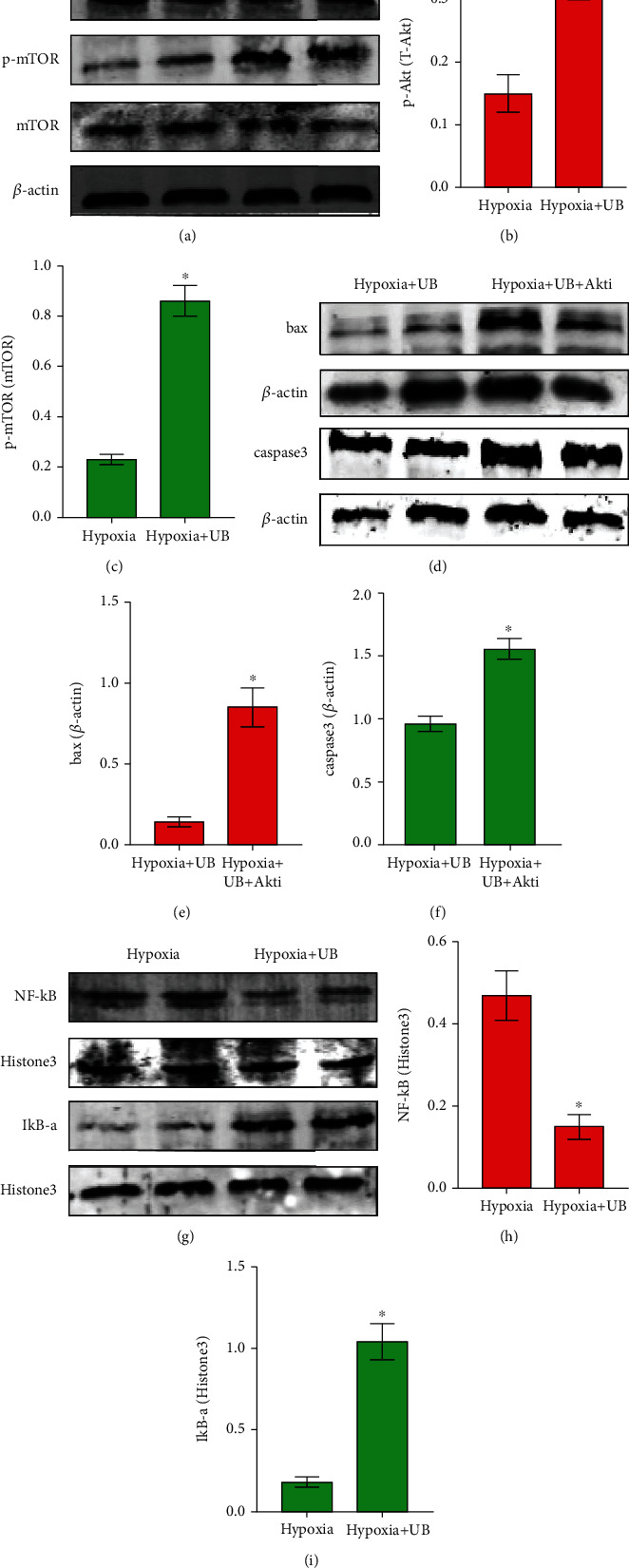
Urolithin B activates the PI3K/Akt pathway to inhibit hypoxia-induced cardiomyocyte apoptosis and suppressed nuclear translocation of NF-*κ*B to facilitate nerve remodeling. (a–c) Western blotting results and densitometric analysis of p-Akt, T-Akt, p-mTOR, and T-mTOR protein levels in the hypoxia group and the hypoxia+urolithin B group. ^∗^*p* < 0.05; *n* = 5 per group (Student's *t*-test). (d–f) Western blotting results and densitometric analysis of bax and caspase-3 protein levels in the hypoxia+urolithin B and hypoxia+urolithin B+Akti group. ^∗^*p* < 0.05; *n* = 5 per group (Student's *t*-test). (g–i) Western blotting results and densitometric analysis of NF-*κ*B and IkBa protein levels in the hypoxia group and the hypoxia+urolithin B group. ^∗^*p* < 0.05; *n* = 5 per group (Student's *t*-test).

To elucidate the underlying mechanisms of urolithin B on cardiomyocytes, we investigated the changes of the components of the apoptosis pathway by the western blotting assay. Zheng et al. identified that urolithin B attenuated cardiomyocyte apoptosis through the Akt/mTOR pathway under hypoxic conditions [2]. We found that urolithin B stimulation significantly increased the phosphorylation of Akt (S473) and mTOR (S2448) (Figures 4(a)–4(c)). Furthermore, urolithin B attenuated the expression of bax and caspase-3 proteins on cardiomyocytes, which was also blocked by an Akt inhibitor (Figures 4(d)–4(f)). Thus, our data suggested that urolithin B prevents cardiomyocyte apoptosis by regulating the Akt/mTOR pathway. Wang et al. revealed the nuclear factor-*κ*B- (NF-*κ*B-) dependent signal pathway involved in cardiac nerve remodeling after MI [12]. MI activated the NF-*κ*B pathway and accelerated cardiac sympathetic hyperinnervation by GAP43 and TH [13]. The western blotting assay indicated that nuclear NF-*κ*B was downregulated with urolithin B treatment. Moreover, nuclear IkBa levels were upregulated, which suggests that urolithin B suppressed nuclear translocation of NF-*κ*B in an IkBa-dependent manner (Figures 4(g)–4(i)). The above results collectively suggest that urolithin B suppressed nuclear translocation of NF-*κ*B to facilitate nerve remodeling and reduced susceptibility to myocardial arrhythmic predisposition.

## 3. Discussion

Our study demonstrated that urolithin B reduced susceptibility to myocardial arrhythmic predisposition after hypoxia, at least in part, via reduction of apoptosis, promotion of nerve remodeling, and inhibition of gap junction channels. In vitro studies showed that urolithin B treatment ameliorates myocardial hypoxia injury. Urolithin B alleviated cardiomyocyte apoptosis via activation of the Akt/mTOR pathway. Moreover, we further revealed that urolithin B suppressed nuclear translocation of NF-*κ*B to facilitate nerve remodeling. Collectively, these findings suggested that urolithin B is a potential therapeutic for cardiac dysfunction and ventricular tachyarrhythmia following ischemic heart disease.

Myocardial apoptosis might be participating in the occurrence of tachyarrhythmia. In fact, Aime-Sempe et al. observed 50 right atrial myocardial specimens from patients with atrial fibrillation and found that most of the specimens had cardiomyocyte apoptosis [6]. Pathologically, apoptosis may cause arrhythmia in the following ways. Firstly, apoptosis contributed to the remodeling of cardiomyocytes, and the changes in the electrophysiological characteristics of the myocardial muscle triggered the frequent recurrence of the tachyarrhythmia. Secondly, in the process of apoptosis, the excitement of cardiomyocytes may increase and even form automatic rhythm points. When agitation reaches that point, anisotropic conduction occurs, which provides the basis for the anatomy of reentrant arrhythmia. The previous study indicated that urolithin B improved the cardiac function by preventing against cardiomyocyte apoptosis [2]. According to our study, we also confirmed that urolithin B inhibited cardiomyocyte apoptosis after hypoxia. Thus, urolithin B can effectively inhibit the formation of arrhythmias.

Cx43 is the predominant ventricular gap junction protein with the function of rapidly spreading and coordinating excitation signals for an effective heart contraction [14, 15]. Thus, it is critical for maintaining normal cardiac electrical conduction [16]. After MI, the reduced expression of Cx43 acts synergistically on conduction abnormalities and reentrant arrhythmias [17–19]. Roell et al. [20] reported that engraftment of Cx43-expressing cells can prevent postinfarct arrhythmia. On the contrary, Lerner et al. [21] demonstrated that in Cx43-defcient mice, the incidence of VAs increased markedly when the coronary artery was occluded. Sympathetic neural remodeling also accounts for the occurrence of arrhythmias after MI. It has been demonstrated that excessive sympathetic activity in the heart can directly lead to arrhythmias in both post-MI patients and animal models [22]. We verified that urolithin B retarded the secretion of inflammatory cytokine, such as IL-6 and TNF-ɑ in our previous research [9]. The previous studies found that IL-6 might promote neural remodeling and inhibit gap junction channel expression [10, 11]. As expected, we observed that urolithin B promoted the expression of Cx43 and inhibited the expression of GAP-43 and TH, markers of nerve remodeling. Therefore, inhibited cardiomyocyte apoptosis, deregulated nerve remodeling, and upregulated gap junction protein may be the primary reasons for UB to reduce susceptibility of arrhythmic predisposition post-MI.

The activated PI3K/Akt pathway, which is a major apoptosis signaling pathway, is involved in MI-induced cardiac dysfunction via affecting cardiomyocyte apoptosis. Cell apoptosis caused by activation of Akt was found in other disease models, including abdominal aortic aneurysm, atherosclerosis, and cardiac hypertrophy. Zheng et al. described that urolithin B protected cardiomyocytes against apoptosis [2]. In the present study, we verified that urolithin B reduced the apoptosis rate of cardiomyocytes, which agrees with other findings. NF-*κ*B is considered to be the upstream of the inflammatory cascade and is involved in the inflammatory response in various cardiac diseases, including MI [12]. Excess inflammatory response is responsible for cardiac nerve sprouting [12]. Previous studies revealed the nuclear NF-*κ*B signal pathway involved in cardiac nerve remodeling after MI [12]. We preliminarily demonstrated that urolithin B treatment induced the downregulation of proinflammatory cytokine, such as TNF-*α* and IL-6, at mRNA and protein levels [9]. Thus, the treatment of urolithin B was closely associated with MI-induced sympathetic nerve sprouting.

We achieved the primary goal of our study; nevertheless, there are still some limitations of our study. Firstly, we did not assess the role of urolithin B in vivo, which may hinder further research on the specific mechanism of urolithin B or limit its application. Secondly, we previously reported that urolithin B suppressed myocardial fibrosis and retarded ventricular tachyarrhythmia, but we are not studying the relationship between ventricular gap junction channel and myocardial fibrosis. Thirdly, cell necrosis, necroptosis, and other potential types of cell death may be more related to arrhythmia after myocardial infarction, which was ruled out from the scenario.

To sum up, our results revealed that urolithin B suppresses cardiomyocyte apoptosis and alters gap junction and neural remodeling levels with MI. Mechanistically, the Akt/mTOR pathway and nuclear translocation of NF-*κ*B were investigated to contribute to the protective effect of urolithin B against cardiomyocyte hypoxia. These results highlight the potential of urolithin B to be used as adjuvant therapy for myocardial infarction.

## 4. Materials and Methods

### 4.1. Myocardial Infarction Model Establishment

C57BL/6 mice were obtained from the Laboratory Animal Center of Nanchang University. All animal experiments were approved by the Animal Research Committee of Nanchang University and performed in accordance with the National Institutes of Health Guide for the Care and Use of Laboratory Animals (no. 2016-0074). Briefly, adult male mice (56-70 days old) were anesthetized by an intraperitoneal injection of 3% pentobarbital sodium (40 mg/kg). The left anterior descending (LAD) coronary artery was ligated with a silk suture, 1 mm from the ascending aorta. In sham-operated animals, an analogous surgical operation was performed without occlusion of the LAD. All mice were sacrificed by cervical dislocation at the indicated time points [13].

### 4.2. Cell Culture, Hypoxia Model, and Chemical Treatment

Cardiomyocytes for the in vitro experiments were isolated from D1 C57BL/6 mice. Neonatal mice were anesthetized using 2% inhaled isoflurane. Isolation and culture of ventricular cardiomyocytes were performed as previously described [13]. Cells were cultured in serum-free, glucose-free, and sodium pyruvate-free Dulbecco's modified Eagle's medium (Invitrogen, Carlsbad, CA) and cultured in an anoxia chamber (In Vivo 500; Ruskinn Life Science) under hypoxia (94% N_2_/5% CO_2_/1% O_2_) for 3 h. Pretreatment with urolithin B (Sigma, US) was performed as described previously [2].

### 4.3. Quantitative Real-Time Reverse Transcription PCR (qRT-PCR)

The TRIzol reagent (Invitrogen) was used to extract total RNA, and complementary DNAs (cDNAs) were synthesized using the PrimeScript RT Reagent Kit (Takara, Otsu, Japan). RT-PCR was performed in a 20 *μ*l reaction system and analyzed by using a SYBR Green RT-PCR Kit (Takara Biotechnology) with the LightCycler 480 II system (Roche Diagnostics, Basel, Switzerland). Glyceraldehyde-3-phosphate-dehydrogenase (GAPDH) mRNA was used as controls. The following primer sequences were used (5′-3′): bcl2 forward: 5′-CTTCGCCGAGATGTCCAG-3′ and reverse: 5′-GGCTCAGATAGGCACCC-A-3′; bax forward: 5′-CCCACCAGCTCTGAACAGTTC-3′ and reverse: 5′-CCAGCCACAAAGATGGTCAC-3′; caspase-3 forward: 5′-CTGAAGGCTCCTGG-TTTA-3′ and reverse: 5′-TGCCACTCTGCGATTTAC-3′; IL-6 forward: 5′-ACTCCATCTGCCCTTCA-3′ and reverse: 5′-ACTCCATCTGCCCTTCA-3′; CX43 forward: 5′-GAGTTTGCCTAAGGCGCT-C-3′ and reverse: 5′-AGGAGTTCA-ATCACTTGGCG-3′; TH forward: 5′-GCGATCAGGCCCAAGATGTA-3′ and reverse: 5′-AATGTATCGAGCCAGGTC-3′; GAP43 forward: 5′-AACGGAGACTG-CAGAAAGC-3′ and reverse: 5′-CCTTAGGTTTGGCTTCGTCT-3′; and GAPDH forward: 5′-TTCAATGGCACAGTCAAGGC-3′ and reverse: 5′-TCACCCCATTT-GATGTTAGCG-3′. Primers were designed using Primer Express® software (Thermo Fisher Scientific, Frankfurter, Germany) with nucleotide sequences obtained from the GenBank database.

### 4.4. Western Blot Analysis

Protein was extracted from cardiomyocytes using lysis buffer containing protease inhibitors and a protein phosphatase inhibitor. Protein concentrations were determined with a bicinchoninic acid (BCA) protein assay kit (Pierce; Rockford, IL, USA). Protein was denatured at 100°C for 10 min. Equal quantities of protein extracts (50 *μ*g) were separated using 10% SDS-PAGE gel and transferred onto a nitrocellulose membrane. Membranes were further probed with primary antibodies against phosphorylated (p)-Akt (1 : 1000; CST #4060), total (t)-Akt (1 : 1000; CST #9272), phosphorylated (p)-mTOR (1 : 1000; CST #5536), total (t)-mTOR (1 : 1000; CST #2972), bcl2 (1 : 1000; CST #3498), bax (1 : 1000; CST #5023), caspase-3 (1 : 1000; CST #14220), Cx43 (1 : 1000; CST #3512), GAP-43 (1 : 1000; CST #8945), TH (1 : 1000; CST #2792), IL-6 (1 : 1000; Abcam #ab233706)), NF-*κ*B (1 : 1000; CST #8242), IkBa (1 : 1000, CST #4812), and *β*-actin (1 : 1000, CST #4970) at 4°C overnight. Then, membranes were incubated with an HRP-labeled secondary antibody in blocking buffer. Bands were detected using an enhanced electrochemiluminescence (ECL) detection kit. Finally, the density of proteins was analyzed using ImageJ software and normalized to *β*-actin.

### 4.5. TUNEL Immunofluorescence Staining

Cardiomyocytes were stimulated with hypoxia or stimulated with hypoxia followed by urolithin B. Then, the cells were fixed with formalin for 1 hour, and apoptotic cells were labeled with a TUNEL kit. Nuclei were stained with DAPI (Millipore, Beverly, MA, USA). The rate of apoptotic cells (positive TUNEL staining cells/DAPI staining cells) in the hypoxia+urolithin B group was compared with that in the hypoxia group. Images were captured under Leica TCS SP8 fluorescence confocal microscopy for further analysis.

### 4.6. Statistical Analysis

All data were analyzed with the statistical software GraphPad Prism 5.0 (GraphPad Software Inc.) and expressed as means ± SEM. The normal distribution was confirmed by the Shapiro-Wilk test (*p* > 0.1) with SPSS 17.0. The differences between each group were analyzed using one-way ANOVA. *p* < 0.05 was considered statistically significant. Further, the Tukey post hoc analysis (*α* = 0.05) was done to confirm where the differences occurred between groups.

## Data Availability

We have not used any publicly archived datasets.
